# Diagnostic performance of circulating biomarkers for non-alcoholic steatohepatitis

**DOI:** 10.1038/s41591-023-02539-6

**Published:** 2023-09-07

**Authors:** Arun J. Sanyal, Sudha S. Shankar, Katherine P. Yates, James Bolognese, Erika Daly, Clayton A. Dehn, Brent Neuschwander-Tetri, Kris Kowdley, Raj Vuppalanchi, Cynthia Behling, James Tonascia, Anthony Samir, Claude Sirlin, Sarah P. Sherlock, Kathryn Fowler, Helen Heymann, Tania N. Kamphaus, Rohit Loomba, Roberto A. Calle

**Affiliations:** 1 Virginia Commonwealth University School of Medicine, Richmond, VA USA; 2 Pfizer, Inc., Sacramento, CA USA; 3 Bloomberg School of Public Health, Johns Hopkins University, Baltimore, MD USA; 4 Cytel, Inc., Waltham, MA USA; 5 P Value Communications, LLC, Cedar Knolls, NJ USA; 6 Saint Louis University, St. Louis, MO USA; 7 Liver Institute Northwest, Seattle, WA USA; 8 Indiana University School of Medicine, Indianapolis, IN USA; 9 Department of Pathology, University of California San Diego School of Medicine, San Diego, CA USA; 10 Center for Ultrasound Research & Translation, Department of Radiology, Massachusetts General Hospital,Harvard Medical School, Boston, MA USA; 11 Deptartment of Radiology, University of California San Diego School of Medicine, San Diego, CA USA; 12 Pfizer, Inc., Boston, MA USA; 13 US Food and Drug Administration, Silver Springs, MD USA; 14 NAFLD Research Center, Division of Gastroenterology, Department of Medicine, University of California, San Diego, La Jolla, CA USA; 15 Regeneron Pharmaceuticals, Inc., Tarrytown, NY USA

**Keywords:** Diagnostic markers, Biomarkers

## Abstract

There are no approved diagnostic biomarkers for at-risk non-alcoholic steatohepatitis (NASH), defined by the presence of NASH, high histological activity and fibrosis stage ≥2, which is associated with higher incidence of liver-related events and mortality. FNIH-NIMBLE is a multi-stakeholder project to support regulatory approval of NASH-related biomarkers. The diagnostic performance of five blood-based panels was evaluated in an observational (NASH CRN DB2) cohort (*n* = 1,073) with full spectrum of non-alcoholic fatty liver disease (NAFLD). The panels were intended to diagnose at-risk NASH (NIS4), presence of NASH (OWLiver) or fibrosis stages >2, >3 or 4 (enhanced liver fibrosis (ELF) test, PROC3 and FibroMeter VCTE). The prespecified performance metric was an area under the receiver operating characteristic curve (AUROC) ≥0.7 and superiority over alanine aminotransferase for disease activity and the FIB-4 test for fibrosis severity. Multiple biomarkers met these metrics. NIS4 had an AUROC of 0.81 (95% confidence interval: 0.78–0.84) for at-risk NASH. The AUROCs of the ELF test, PROC3 and FibroMeterVCTE for clinically significant fibrosis (≥stage 2), advanced fibrosis (≥stage 3) or cirrhosis (stage 4), respectively, were all ≥0.8. ELF and FibroMeter VCTE outperformed FIB-4 for all fibrosis endpoints. These data represent a milestone toward qualification of several biomarker panels for at-risk NASH and also fibrosis severity in individuals with NAFLD.

## Abstract (web summary)

The NIMBLE biomarker qualification project evaluated the diagnostic performance of five blood-based biomarkers in a cohort of individuals with non-alcoholic fatty liver disease and showed good discriminative performance over commonly used laboratory tests in detecting various stages of non-alcoholic steatohepatitis progression.

## Main

Non-alcoholic fatty liver disease (NAFLD) is a leading cause of liver-related morbidity and mortality^1^. The presence of non-alcoholic steatohepatitis (NASH), an active form of NAFLD, and liver fibrosis stage 2 or higher is linked to an increased incidence of liver-related adverse clinical outcomes and death and is also referred to as ‘at-risk’ NASH^2–4^. Identification of individuals with at-risk NASH for therapy is a cornerstone for clinical care and inclusion in therapeutic trials^5^.

Histological evaluation of liver biopsy sections is the reference standard for diagnosis of NASH as well as quantification of disease activity and fibrosis stage, but it requires an invasive liver biopsy with its associated risks and limitations, hindering its widespread use^6–8^. This has spurred much work to establish non-invasive tests (NITs) to diagnose NASH and fibrosis, yet none has met the evidentiary requirements needed for regulatory qualification. The lack of regulatory approval limits availability of these tests for widespread clinical use. It also hinders patient recruitment into clinical trials and their further development for treatment response monitoring. From a public health point of view, the lack of approved biomarker panels for diagnostic purposes is, thus, a major barrier to access to care and drug development^9^. Although progress has been made in retrospective comparative assessment of NITs^10^, evidence gaps remain for full qualification. Development of such NITs to regulatory standards remains a major unmet need for the field.

There are three general pathways for regulatory approval of biomarker panels^11^. Drug development tools are developed and validated in the context of a specific drug development program, and the approval is limited in a narrowly defined context of use. Academic consensus is another pathway, but it is limited by lack of standardized reported outcomes and publications based on studies that are not designed to meet typical regulatory standards. Biomarker qualification is the third pathway. It is a process wherein regulatory agencies agree that a given biomarker, when used in a specific clinical setting to answer a specific question, provides actionable information with a prespecified level of certainty^11^.

Regulators recognize collaborative initiatives and consortia as a vehicle to tackle the qualification process^12^. It includes consideration of the analytic robustness of the assay and clear definition of the clinical settings and boundaries within which the biomarker assays work. It further requires rigorous assessment of sensitivity and specificity for its intended use and validation across relevant populations. Finally, it also includes an assessment of benefit versus the risks of misclassification. The overall use case is defined by the context of use, which defines who the test will be used on and the clinical setting where it will be used, the purpose of the test, the read-out and its interpretation and the decisions that will emanate from the read-out. The purpose can be diagnostic, prognostic, predictive, disease monitoring or assessment of treatment response^13^. Together, this represents a substantial amount of data, which, for practical logistic reasons, are usually generated in a multi-step manner.

The Foundation for the National Institutes for Health (FNIH) was established by the federal government of the United States as a platform to enable public–private partnerships, bringing multiple federal agencies together with academics, industry partners and other relevant stakeholders to solve problems of great public health importance. The Non-Invasive Biomarkers for Metabolic Liver Disease (NIMBLE) project was commissioned by the FNIH to qualify NITs for NAFLD^9^. It represents a collaborative effort involving the FNIH, the US Food and Drug Administration (FDA), academics and 14 industry partners to qualify biomarkers for diagnostic enrichment of ‘at-risk’ NASH and its subcomponents. NIMBLE has an imaging workstream and a circulating biomarker workstream. The current study is the final report of stage 1 of the NIMBLE project’s circulating biomarker workstream and represents a collaboration between the NIMBLE circulating biomarker workstream and the adult clinical centers and the data coordinating center of the NASH Clinical Research Network (NASH CRN) of the National Institute of Diabetes and Digestive and Kidney Diseases (NIDDK) (Extended Data Table 1). It evaluates the performance metrics of several biomarker panels for the diagnosis of NASH, at-risk NASH and varying severity of fibrosis in individuals with NAFLD. The results of this study will inform if any of the biomarkers have met the evidence needed for qualification or if they are supportive but need additional validation in stage 2. They will also inform if any of these are not considered for final validation efforts in stage 2 of the NIMBLE project.

The first step in the biomarker qualification path is regulatory acceptance of a letter of intent establishing the scientific roadmap to be taken. A NIMBLE study letter of intent for the circulating biomarkers in the current study has been accepted by the FDA, a critical step in the qualification process^14^. The proposed context of use was for diagnostic enrichment for at-risk NASH and its components—that is, presence of NASH, high histological disease activity and specific fibrosis thresholds, for example clinically significant fibrosis (≥stage 2), advanced fibrosis (≥stage 3) or cirrhosis (stage 4), in a population with NAFLD or risk factors for NAFLD. A successful diagnostic enrichment biomarker is expected to select for patients with a higher likelihood of meeting the criteria for at-risk NASH in a subsequent liver biopsy, thus reducing the number of patients who undergo this procedure unnecessarily and improving the efficiency of the process to select patients in need of clinical intervention and/or suitable for participation in NASH clinical trials.

The panels chosen represent the first wave of circulating biomarkers going through this qualification effort and included NIS4 (Genfit, Lille, France), OWLiver (One Way Lipidomics, Bilbao, Spain), PROC3 (Nordic Bioscience, Copenhagen, Denmark), enhanced liver fibrosis (ELF) (Siemens Healthineers, New Jersey, USA) test and the FibroMeter VCTE (Echosense, Paris, France). The latter was chosen as the best vibration controlled transient elastography (VCTE)-linked panel at the time when NIMBLE was designed^15^. Since the design of NIMBLE, additional panels have emerged—for example, the FAST, Agile and ADAPT scores; the qualification of these is expected to follow the roadmap established by the letter of intent for NIMBLE^10,16,17^. A separate qualification effort for the FAST score is underway already.

The goal of the current step in the qualification process was not to identify novel biomarkers or to determine which biomarker is the best but to rigorously determine the sensitivity and specificity of each of the biomarker panels in a curated cohort with a balanced distribution of fibrosis stage and to compare their performance to commonly used laboratory tests for the same purpose used by the general medical community. The data would inform the further development of the selected biomarkers and provide the foundation for a full qualification plan for these panels which, after acceptance by the FDA, will support their qualification with or without additional data from stage 2 as a final step. This critical step thus moves the field closer to having qualified NITs that can be used to identify individuals with at-risk NASH and its subcomponents for both routine practice and drug development.

## Results

**Fig. 1:**
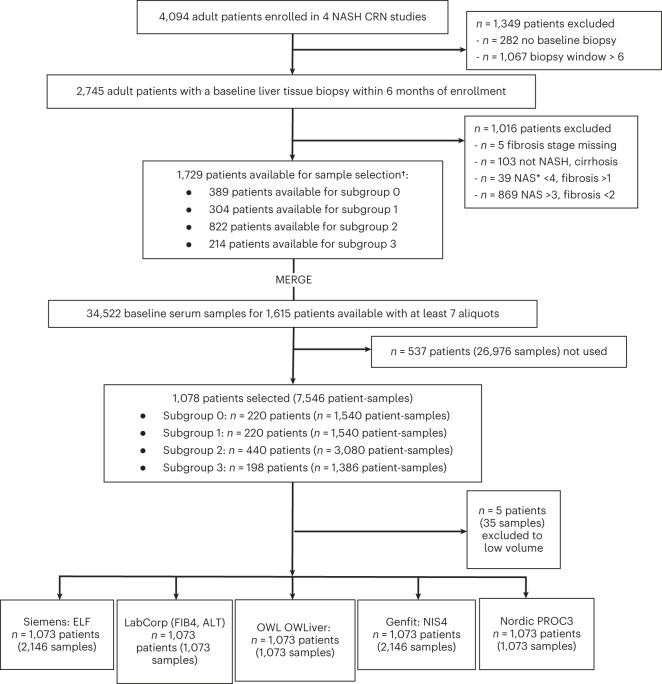
Study population derivation. Sample derivation from the NASH CRN cohort and their use for laboratory analysis of the components of NIS4, OWLiver, PROC3, ELF test and FibroMeter VCTE.

The current study evaluated the diagnostic performance of five biomarker panels (NIS4, OWLiver, PROC3, ELF and FibroMeter VCTE) for the diagnosis of NASH, high NAFLD activity score or varying severity of hepatic fibrosis in a population with NAFLD (Extended Data Table 2). The study cohort was derived from the NASH CRN study cohort, which had 4,094 participants (Fig. 1). A total of 2,479 individuals were excluded because of age, lack of samples or lack of evaluable liver biopsies. Of the remaining individuals, consecutive patients for each stage of disease were selected to ensure that enough patients were available to meet sample size estimates and to have a relatively balanced-distributed spectrum of fibrosis severity (stages 0: *n* = 222; stage 1: *n* = 114; stage 2: *n* = 262; stage 3: *n* = 277; and stage 4: *n* = 198). A total of 1,073 individuals meeting the eligibility criteria were, thus, included for this analysis (Table 1). The mean time from blood sample to biopsy varied from 55 d to 79 d with s.d. of about 24–25 d for most groups except cirrhosis where it was 39 d; 946 of 1,073 (88.2%) individuals had blood samples within 90 d of biopsy, and all had samples within 180 d of biopsy.

**Table 1:** Demographic, clinical and laboratory data from the study cohort

	Stage 0	Stage 1	Stage 2	Stage 3	Stage 4
	*n* = 222	*n* = 114	*n* = 262	*n* = 277	*n* = 198
Age (years)	47.8 (12.2)	48.1 (13.8)	51.7 (11.5)	54.4 (11.2)	56.2 (9.8)
Gender (males) (*n* (%))	99 (44.6%)	52 (45.6%)	102 (38.9%)	91 (32.9%)	60 (30.3%)
White (*n* (%))	158 (71.2%)	68 (59.6%)	199 (76.2%)	217 (78.9%)	169 (86.2%)
African American (*n* (%))	4 (1.8%)	5 (4.4%)	8 (3.1%)	8 (2.9%)	3 (1.5%)
Hispanic (*n* (%))	34 (15.3%)	20 (17.5%)	28 (10.7%)	24 (8.7%)	16 (8.2%)
Other (*n* (%))	26 (11.7%)	21 (18.4%)	26 (10.0%)	26 (9.5%)	8 (4.1%)
Body mass index (kg m^−^^2^)	32.8 (6.6)	33.3 (6.1)	34.5 (6.3)	36.1 (6.6)	36.4 (7.3)
Waist circumference (cm)	104.7 (14.7)	107.3 (13.9)	110.8 (14.2)	114.3 (14.8)	113.7 (15.1)
Type 2 diabetes (*n* (%))	45 (20.3%)	41 (36.0%)	113 (43.1%)	162 (58.5%)	129 (65.2%)
Hypertension (*n* (%))	94 (42.3%)	65 (57.0%)	164 (62.6%)	191 (69.0%)	132 (66.7%)
AST (IU L^−1^)	27.8 (13.3)	31.9 (17.7)	50.3 (29.3)	58.3 (39.8)	51.9 (28.9)
ALT (IU L^−1^)	38.5 (25.4)	45.0 (34.6)	65.5 (43.1)	68.1 (47.8)	49.1 (34.5)
ALP (IU L^−1^)	86.6 (30.5)	80.6 (28.2)	87.0 (28.0)	93.0 (33.2)	114.5 (53.2)
Total bilirubin (mg dl^−1^)	0.5 (0.3)	0.6 (0.5)	0.5 (0.3)	0.5 (0.4)	0.8 (0.8)
INR	1.0 (0.1)	1.0 (0.2)	1.0 (0.1)	1.1 (0.1)	2.8 (4.3)
Albumin (g dl^−1^)	4.6 (0.3)	4.6 (0.3)	4.6 (0.3)	4.5 (0.3)	4.3 (0.4)
Hemoglobin (g dl^−1^)	14.4 (1.4)	14.3 (1.5)	14.2 (1.5)	13.9 (1.5)	13.6 (1.6)
White blood cells (10^3^ per µl)	7.0 (2.2)	7.1 (2.0)	7.3 (2.0)	7.6 (6.0)	6.5 (3.3)
Platelets (cells per ml)	250.3 (62.7)	243.5 (82.9)	237.8 (60.5)	218.7 (66.2)	165.9 (64.1)
Fasting glucose (mg dl^−1^)	101.3 (33.8)	106.7 (42.0)	114.6 (42.2)	116.6 (34.8)	126.2 (51.7)
Fasting insulin (µU ml^−1^)	17.8 (14.2)	25.5 (32.4)	26.8 (29.1)	30.9 (27.6)	35.5 (35.4)
Total cholesterol (mg dl^−1^)	193.7 (43.1)	181.2 (43.3)	189.7 (48.5)	183.3 (42.3)	174.2 (40.4)
LDL-C (mg dl^−1^)	117.5 (36.5)	105.9 (36.6)	112.0 (39.2)	106.1 (38.1)	100.7 (35.3)
HDL-C (mg dl^−1^)	45.1 (10.9)	44.4 (13.5)	42.9 (11.9)	42.8 (11.9)	45.2 (13.2)
Triglycerides (mg dl^−1^)	169.8 (108.0)	168.1 (108.5)	203.1 (275.0)	186.3 (114.9)	141.5 (66.2)
Statin use (*n* (%))	63 (28.4%)	46 (40.4%)	91 (34.7%)	113 (40.8%)	84 (42.4%)
Time from biopsy to study entry (days)	55.16 (24.32)	60.44 (26.93)	53.60 (25.24)	53.18 (24.15)	79.31 (39.22)
NAFL (*n* (%))	195 (87.8%)	23 (20.2%)	0 (0.0%)	0 (0.0%)	7 (3.5%)
NASH (*n* (%))	27 (12.2%)	91 (79.8%)	262 (100%)	277 (100%)	178 (89.9%)
Steatosis grade	1.3 (0.5)	1.2 (0.4)	2.0 (0.8)	1.8 (0.9)	1.3 (0.9)
Ballooning grade	0.1 (0.3)	0.3 (0.5)	1.2 (0.7)	1.5 (0.7)	1.5 (0.7)
Lobular inflammation	1.0 (0.3)	1.1 (0.3)	1.7 (0.7)	1.8 (0.7)	1.5 (0.7)
Portal inflammation	0.8 (0.6)	0.9 (0.5)	1.2 (0.5)	1.4 (0.5)	1.7 (0.5)
NAS	2.5 (0.6)	2.5 (0.6)	4.8 (1.5)	5.2 (1.6)	4.2 (1.6)

All statistics presented are means (s.d.), unless otherwise specified.

*Time between the liver biopsy and study enrollment for 109 (10%) individuals of the cohort was 92–183 d.

The mean age of the cohort was 52.5 years and included 62.3% females. In total, 225 individuals had NAFL present; 835 had NASH; and 13 had cirrhosis with an indeterminate NAFLD phenotype. Those without fibrosis were younger, had mainly fatty liver and not steatohepatitis. They also had a lower NAFLD activity score compared to those with fibrosis stage 2 or higher. The study population for FibroMeter VCTE was a smaller subset of the larger population (*n* = 396) as this analysis was limited to individuals who had a VCTE examination within 6 months of the liver biopsy. The baseline features of this subset were similar to the larger cohort (Extended Data Table 3).

### At-risk NASH

At-risk NASH was defined as presence of steatohepatitis with an NAFLD activity score ≥4 and fibrosis stage ≥2 (refs. ^9,18^). This is correlated with a higher risk of liver outcomes and is the target population for most clinical trials^4^. The prespecified analytic approach for this study was, first, to establish that the area under the receiver operating characteristic curve (AUROC) was at least 0.7 and superior to the unit line—that is, the 95% confidence limits did not intersect 0.5. Second, it was to establish superiority over alanine aminotransferase (ALT), a universally used measure of liver injury for over five decades, for biomarkers intended to assess disease activity and FIB-4 for biomarkers intended to evaluate fibrosis. These were selected because of the amount of pre-existing literature on these biomarkers at the time when NIMBLE was conceived^19,20^ and their wide availability for use by the medical community^21^. The FIB-4 test also provides prognostic information with a step-wise increase in mortality from 0.07 to 0.3 to 2.5 per 100 person-years in individuals with FIB-3 <1.3, 1.3–2.6 and >2.6, further supporting its use as a comparator^22,23^. Other markers for liver injury, such as CK18, were not considered as comparators because they are not universally available or used by the general medical community to assess liver injury. Superiority over ALT and FIB-4 were considered a pragmatic initial step to move to final qualification; biomarker panels that could not meaningfully outperform such simple laboratory measures to inform decision-making would not be suitable for further qualification studies.

**Fig. 2:**
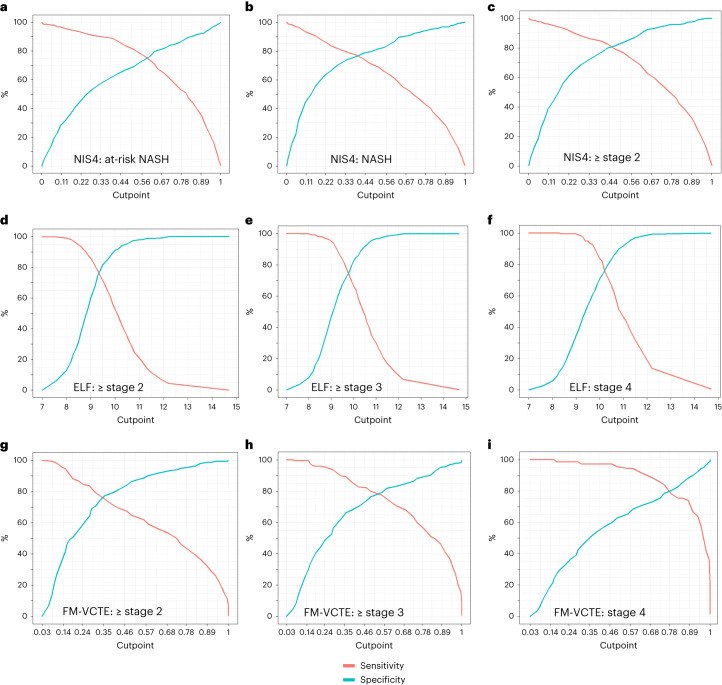
Performance of selected biomarker panels for their intended uses. Sensitivity and specificity of key NIT panels for their respective intended uses are shown as a function of the cutoff scores for the NIT. **a**–**c**, The top panels demonstrate changes in sensitivity and specificity at varying NIS4 cutoff scores for the diagnosis of at-risk NASH (**a**) and its key subcomponent diagnosis of NASH (**b**) and stage 2 or greater fibrosis (**c**). **d**–**f**, The middle panels show similar data for the ELF test for the diagnosis of ≥stage 2 fibrosis (**d**), ≥stage 3 (**e**) and stage 4—that is, cirrhosis (**f**). **g**–**i**, The lower panels demonstrate the changes in sensitivity and specificity at varying FibroMeter VCTE (FM-VCTE) score cutoffs for the diagnosis of ≥stage 2 fibrosis (**g**), ≥stage 3 fibrosis (**h**) and stage 4 fibrosis (**i**). Individual plots were derived from 50 individual score cutoffs covering the range where sensitivity was 100% to where specificity approached 100%, followed by smoothening of the graph to cover the dynamic range of scores for their intended uses.

NIS4 was the only panel with an intended use to diagnose the underlying composite phenotype of at-risk NASH (*n* = 539 within the full cohort). The sensitivity and specificity of NIS4 for this diagnosis were 78.1% and 73.6%, respectively, with an AUROC of 0.815 at the optimal cutpoint (Table 2), which was superior to both ALT (AUROC = 0.726) and FIB-4 (AUROC = 0.704) (*P* < 0.001 NIS4 versus both) (Table 3). The sensitivity and specificity at varying cutpoints along the dynamic range of scores for NIS4 are shown graphically in Fig. 2.

**Table 2:** Sensitivity and specificity of individual panels for their intended use

	Sensitivity (%)	Specificity (%)	Youden index	AUROC	Significance
				(95% CI)	(versus ALT or FIB4)
NASH diagnosis					
ALT	63.2	64.8	0.28	0.678 (0.639, 0.717)	
NIS4	77.7	76.2	0.539	0.832 (0.801, 0.864)	<0.001
OWL	77.3	66.8	Categorical data AUROC cannot be computed		
NAS ≥4					
ALT	71.1	64.1	0.352	0.726 (0.694, 0.759)	
NIS4	78.1	73.6	0.517	0.815 (0.786, 0.844)	<0.001
At-risk NASH					
ALT	71.1	64.1	0.352	0.726 (0.694, 0.759)	
FIB4	76.4	58.4	0.349	0.704 (0.671, 0.737)	
NIS4	78.1	73.6	0.517	0.815 (0.786, 0.844)	<0.001
Fibrosis stage ≥2					
FIB4	65.6	80.6	0.462	0.798 (0.768, 0.828)	
ELF	71.8	81.5	0.533	0.828 (0.08, 0.857)	0.013
NIS4	82.3	79.9	0.622	0.874 (0.848, 0.899)	<0.001
PROC3	69.8	81	0.507	0.809 (0.779, 0.839)	0.279
FibroMeter VCTE	66.7	86.4	0.53	0.841 (0.796, 0.886)	<0.001
Fibrosis stage ≥3					
FIB4	70.3	72.4	0.427	0.789 (0.758, 0.819)	
ELF	80.8	70.2	0.509	0.835 (0.807, 0.863)	<0.001
NIS4	72.9	74.8	0.476	0.788 (0.757, 0.820)	0.615
PROC3	71.4	71.4	0.428	0.764 (0.732, 0.795)	0.947
FibroMeter VCTE	76.2	81.3	0.575	0.858 (0.814, 0.902)	<0.001
Fibrosis stage 4					
FIB4	84.7	62.9	0.476	0.810 (0.770, 0.850)	
ELF	82.1	73.3	0.555	0.855 (0.818, 0.892)	<0.001
NIS4	78.1	61.4	0.395	0.725 (0.681, 0.760)	1
PROC3	66.2	68.5	0.346	0.728 (0.685, 0.770)	1
FibroMeter VCTE	94.2	70.4	0.646	0.897 (0.843, 0.951)	0.002

**Table 3:** Performance of biomarkers at high sensitivity and specificity

	When constraining sensitivity to be at least 90%			When constraining specificity to be at least 90%		
	Cutpoint	Specificity (%)	Significance	Cutpoint	Sensitivity (%)	Significance
NASH diagnosis						
ALT	≥22.0	26.9		≥72.0	26.3	
NIS4	≥0.20	55.9	<0.001	≥0.7	54.2	<0.001
NAS ≥4						
ALT	≥25.0	28.1		≥73.0	32.3	
NIS4	≥0.30	57.8	<0.001	≥0.80	46.2	<0.001
At-risk NASH						
ALT	≥23.0	25.7		≥ 73.0	27.1	
FIB4	≥ 0.8	44.0		≥ 1.7	46.1	
NIS4	≥0.2	64.4	<0.001	≥0.6	67.2	<0.001
Fibrosis (fibrosis stage ≥2)						
FIB4	≥0.8	44		≥1.7	46.1	
NIS4	≥0.2	64.4	<0.001	≥0.6	67.2	<0.001
ELF	≥8.8	48.7	0.013	≥10.0	52.8	0.013
PROC3 (ELISA)	≥12.8	36.3	0.279	≥20.1	46.7	0.279
FibroMeter VCTE	≥0.2	50	<0.001	≥0.6	60.2	<0.001
Advanced fibrosis (fibrosis stage ≥3)						
FIB4	≥1.0	43.7		≥2.1	43.6	
NIS4	≥0.3	49.7	0.615	≥0.9	37	0.615
ELF	≥9.2	55.3	<0.001	≥10.4	50.3	<0.001
PROC3	≥13.6	34.6	0.947	≥25.0	42.5	0.947
Fibrometer VCTE	≥0.3	59.6	<0.001	≥0.8	54.2	<0.001
Cirrhosis (fibrosis stage 4)						
FIB4	≥1.3	50.5		≥2.6	42.3	
NIS4	≥0.5	46	1	≥0.9	23	1
ELF	≥9.7	60.5	<0.001	≥10.9	49	<0.001
PROC3	≥15.1	37.3	1	≥30.6	29.8	1
FibroMeter VCTE	≥0.7	72.5	0.002	≥0.9	66.7	0.002

Note: The *P* values reflect comparisons of performance between individual panels versus ALT for diagnosis of NASH or NAS ≥4 and to FIB-4 for diagnosis of fibrosis categories or both for at-risk NASH.

### NASH diagnosis

NIS4 and the OWLiver tests had an intended use to diagnose NASH (Extended Data Table 2). NIS4 (Youden cutpoint 0.539) had an AUROC of 0.83 (95% confidence interval (CI): 0.8–0.86) and was superior to ALT (AUROC = 0.67) for this intended use (Table 2). The sensitivity and specificity were 77.7% and 76.2%, respectively, at this cutpoint. NIS4 had a specificity of 47.7% and a sensitivity of 54.4% when sensitivity and specificity were constrained at 90%, respectively (Table 3). Under both conditions with either sensitivity or specificity constrained at 90%, NIS4 was significantly superior to ALT (*P* < 0.001 for both). OWLiver provided the results in categorical format, which did not permit generation of an AUROC; it diagnosed NASH with a sensitivity of 77.3% and a specificity of 66.8%.

### High NAFLD activity score (≥4)

A high NAFLD activity score (NAS) is a component of at-risk NASH. The AUROC (0.815, 95% CI: 0.786–0.844) for NIS4 was significantly superior to ALT (AUROC: 0.726, sensitivity 71.1%, specificity 64.1%), the comparator for panels intended to diagnose high activity (*P* < 0.001). The specificity and sensitivity of NIS4 were 57.8% and 46.2%, respectively, when sensitivity and specificity were locked at 90%. Under both conditions, the diagnostic performance of NIS4 was significantly superior to ALT (*P* < 0.001 for both) (Table 3).

### Clinically significant fibrosis (fibrosis stage ≥2)

NIS4, ELF, PROC3 and FibroMeter VCTE had an intended use to identify clinically significant fibrosis in individuals with NAFLD. The AUROCs were as follows: NIS4 (0.874), ELF (0.828), PROC3 (0.8) and FibroMeter VCTE (0.841). Their respective sensitivity and specificity at their Youden cutoff are provided in Table 2. FIB-4 had an AUROC of 0.798, which was very close to the expected benchmark AUROC of 0.8 (ref. ^19^). NIS4 (*P* < 0.001), ELF (*P* < 0.01) and FibroMeter VCTE (*P* < 0.001) were all significantly superior to FIB-4. However, the overall AUROC for PROC3 was not superior to FIB-4. Similar data were obtained when the performance of these panels with sensitivity and specificity constrained at 90% were evaluated (Table 3).

### Advanced fibrosis (stage ≥3)

The operational definition of advanced fibrosis included individuals with stage 3 or 4. The AUROCs of the panels tested for the diagnosis of advanced fibrosis were as follows: FIB-4 (0.789), ELF (0.835, *P* < 0.001 versus FIB4), PROC3 (0.809, *P* = not significant (NS) versus FIB-4) and FibroMeter VCTE (0.841, *P* < 0.001 versus FIB4). A secondary analysis of NIS4 for advanced fibrosis provided an AUROC of 0.78 (*P* = NS versus FIB4). The sensitivity with specificity fixed at 90% were 50.3% and 54.2%, whereas the specificity was 55.3% and 59.6% with sensitivity fixed at 90% for ELF and FibroMeter VCTE, respectively; for both analyses, ELF and FibroMeter VCTE were superior to FIB-4 (*P* < 0.001 for both biomarkers for both analyses) (Table 3).

### Cirrhosis (stage 4)

The AUROCs for the diagnosis of cirrhosis were 0.81 for FIB-4, 0.855 for ELF (*P* < 0.001 versus FIB-4) and 0.897 for FibroMeter VCTE (*P* = 0.002 versus FIB-4). The sensitivity of ELF and FibroMeter VCTE at the Youden cutpoint were 82.1% and 94.2%, and the specificities were 73.3% and 70.4%, respectively. Their performance at 90% sensitivity (specificity: ELF 60.5%, FibroMeter VCTE 72.5%) and 90% specificity (sensitivity: ELF 49%, FibroMeter VCTE: 66.7%) were also significantly superior to FIB-4 (ELF: *P* < 0.001 for both analyses, FibroMeter VCTE: *P* = 0.002 for both analyses) (Table 3).

## Discussion

The current study demonstrates that NIS4 met the prespecified criteria for further qualification efforts for diagnostic enrichment for NASH, for high NAS and for at-risk NASH. Also, the ELF test and FibroMeter VCTE met the criteria for further qualification efforts for the diagnosis of clinically significant fibrosis (≥stage 2), advanced fibrosis (≥stage 3) and cirrhosis (stage 4) in individuals with NAFLD. These data inform the development and approval of the full qualification plan that will define the final set of studies needed for regulatory approval for diagnostic enrichment tools for NASH^14^.

This study has implications for the approval of biomarkers for diagnostic evaluation of at-risk NASH and its components. The definition of the sensitivity and specificity for each biomarker for each of its intended uses in individuals with NAFLD sets the stage for its validation in other cohorts, retrospective and prospective, with varying prevalence of each phenotype evaluated as the final step for qualification. The current study was a first step to determine if the biomarker panels not only identified the relevant phenotypes based on their intended use but also if they were superior to some commonly used clinical laboratory tools, such as ALT and FIB-4. These will serve as criteria, to be finalized with feedback from the FDA, to move the panels with the most promising performance metrics to the final qualification steps.

Another implication of the current study is that, along with the approved letter of intent, it establishes a roadmap for qualification of biomarkers for diagnostic enrichment. This regulatory roadmap is also likely to be used by other panels, such as FAST, ADAPT and Agile, that were developed after NIMBLE was initiated and are also strong candidates to be moved forward for qualification. The establishment of qualified biomarkers for diagnostic enrichment will also set the stage for their use for disease monitoring and treatment response biomarkers, which will be critically needed to establish a surrogate endpoint based on NITs alone. In this respect, the diagnosis of cirrhosis is particularly relevant because progression to cirrhosis as assessed histologically is already a generally accepted surrogate endpoint to assess therapeutic efficacy^5^.

The practical application of these data has to be considered in the context of how the tests are used (Extended Data Tables 4 and 5). In primary care, where the prevalence of advanced fibrosis is 1%, positive tests are likely to be false positives, and, even with excellent sensitivity and specificity, the positive predictive value (PPV) will be low^24^. Using these tests to identify patients for clinical trials in such settings is likely to have many false positives, resulting in high screen fail rates. The negative predictive value (NPV) for FIB4, as well as all of the biomarker panels evaluated, ranged from 98% to 99.7% when the population prevalence of advanced fibrosis was 1% (Extended Data Table 4). These tests can, therefore, be applied for exclusion of this phenotype for both clinical management and to exclude individuals during screening for clinical trials targeting individuals with at-risk NASH, particularly in a primary care setting.

The prevalence of at-risk NASH or its subsets, NASH with advanced fibrosis or cirrhosis are higher in hepatology clinics and range from 10% to 40%^2,25,26^. The high NPV in settings with low prevalence was maintained at these ranges, whereas the PPVs approached 80% at the 40% prevalence when the Youden cutpoint was used (Extended Data Table 5). In clinical trial settings, these data should allow exclusion of individuals without these phenotypes while limiting overdiagnosis compared to a primary care setting. Additional enhancement of certainty for ruling in disease by using the cutpoint for 90% specificity (Table 3) will, however, be associated with a loss of sensitivity and increased potential for misclassification.

Further improvement is likely to require an algorithmic approach using multiple panels or use of imaging-based tests for greater precision in identification of this population. Magnetic resonance elastography with FIB4 or aspartate transaminase (AST) has been shown to identify individuals with NASH and advanced fibrosis or at-risk NASH, respectively, and may provide such tools^27–29^. The current data cannot, however, be directly compared to these due to methodological differences.

For patients with advanced fibrosis or cirrhosis, a mistaken diagnosis of absence of these phenotypes may cause them to be followed without surveillance for hepatocellular cancer or gastro-esophageal varices, which are needed for those with cirrhosis. The overall high NPVs suggest that the risks are, in general, low. Conversely, overdiagnosis due to modest PPVs may result in redundant additional testing, including liver biopsy with its associated risks. ELF and FibroMeter VCTE can identify 82–94% of true-positive cases of cirrhosis but may also overdiagnose some patients for cirrhosis in clinics with high prevalence of cirrhosis (Extended Data Table 5). The risks of overdiagnosis have to be considered in the context of the risks of missing advanced fibrosis or cirrhosis altogether in specific populations, both in clinical practice and for consideration for inclusion in trials.

This study has several methodological strengths. The time from biopsy to blood draw was short, and all analyses, including the comparators, were made using the same blood sample. Furthermore, all samples were drawn, aliquoted, stored and analyzed without multiple freeze–thaw cycles using prespecified protocols and verifiable chain of custody. All laboratory tests were run contemporaneously on these samples. Histology was read independently using a rigorous prespecified protocol by the pathology committee of the NASH CRN masked to clinical and laboratory data^30,31^. The distribution of fibrosis stages in the cohort was balanced and, thus, avoided fibrosis-related spectrum bias. Finally, for each of the phenotypes studied—for example, NASH or NAS ≥4 or varying fibrosis cutoffs—the sample size included enough individuals with and without the phenotype to assure power for both sensitivity and specificity. The chain of custody of sample from withdrawal from the NIH biorepository to its analysis in individual laboratories and the subsequent data transfer to the NIMBLE data coordinating center and integration with metadata followed a prespecified and verifiable protocol to ensure data and the overall integrity of the project.

This study also has some limitations. The NASH CRN is based at tertiary care centers, generating ascertainment bias. The study population was also predominantly White ethnicity, and the data are not generalizable to other ethnicities. The curated patient population to ensure a balanced distribution of fibrosis stages to rigorously define sensitivity and specificity did not allow evaluation of the predictive values in populations with variable distribution of disease phenotypes. This will be performed in the final qualification step, and the current study sets the stage for the evaluation of these diagnostic cutoffs to be validated in these future analyses. Another potential limitation is that new biomarkers—for example, FAST, Agile and ADAPT—are not studied in the predetermined qualification panel. These were, however, not developed at the time the current study was conceived, and they are currently undergoing rigorous evaluation and will be reported as post hoc analyses separately. Furthermore, although the study population was specifically curated to have a relatively even distribution of fibrosis stages to avoid spectrum bias, real-world populations do not have such a distribution, and the PPV and NPV of the tests in populations with varying prevalence may require separate confirmation. It must, however, be noted that the journey from discovery and initial validation of a biomarker to a diagnostic tool that is approved for use by all clinicians is a long one and involves many steps that cannot be combined in one study.

In conclusion, multiple biomarker panels met the prespecified criteria described in the letter of intent for biomarker qualification by the FDA in stage 1 of the circulating workstream of the NIMBLE project of the FNIH. These findings inform the development of the full qualification package for these biomarkers for diagnostic enrichment in the next stage of the NIMBLE project.

## Methods

Serum samples collected from adult participants with NAFLD in a non-interventional registry (database 1 and database 2 (DB1 and DB2)) and baseline samples from clinical trials (PIVENS and FLINT) across 12 NIDDK NASH CRN clinical sites (Extended Data Table 1) were analyzed. The investigators have analyzed the data and take responsibility for the contents of this manuscript. The studies were done in accordance with STARD guidance and reported using the TRIPOD statement^32,33^.

### Ethics statement

This study was approved as an ancillary study of the NASH CRN, and the study samples were curated from the NASH CRN biorepository that was linked to the DB1 and DB2 registry studies and from baseline samples from the PIVENS and FLINT trials (ClinicalTrials.gov: 01030484, 01265498 and 00063622). It is a post hoc analysis of samples and clinical–histological data from selected individuals from these studies who met the criteria for the current study. These studies were approved by the individual site institutional review boards (IRBs) for these studies, and all patients provided informed consent, including the use of their blood samples for additional analyses. The current study was performed on a de-identified clinical dataset and on blood samples of such individuals who had blood samples drawn and frozen within 180 d of a liver biopsy. A list of individual site IRB approvals is provided as Supplementary Table 1.

### Context of use

In individuals with NAFLD or with risk-factors for NAFLD, to serve as a diagnostic enrichment tool for the identification of various histological phenotypes of NAFLD, intended for selection for participation in NAFLD/NASH clinical trials and/or drug treatment. Individuals who were overweight or obese, or who had other features of metabolic syndrome, were considered to be at risk for NAFLD^25^. The presence of specific phenotypes to be diagnosed included:

- At-risk NASH: (NASH + NAS ≥4 + fibrosis stage 2 or higher)
- NASH (borderline or definite)
- NAS ≥4
- Clinically significant fibrosis (fibrosis stage ≥2)
- Advanced fibrosis (stages 3 or 4)
- Cirrhosis (stage 4)

### Study design

#### Study population

The study population was curated from the CRN patient base to ensure sufficient number of individuals with and without the histological phenotypes of interest and a balanced distribution of fibrosis stages to avoid fibrosis spectrum bias. These included patients with biopsy-proven NAFLD who had stored serum obtained within 180 d of a liver biopsy. Patients were derived from four different NASH CRN studies; these included the non-interventional registry studies (DB1 and DB2) and the PIVENS and FLINT clinical trials. The results of the clinical trials were previously published. For patients in DB2, baseline biopsy and serum samples were used from the time of entry, whereas, for DB1, biopsies performed both at baseline and during follow-up were considered. For the clinical trials, only baseline samples and biopsy data before randomization were included. The liver biopsies had already been read and scored by the pathology committee of the NASH CRN using previously published methods^23,30^. The histological data from the CRN database were used for this analysis.

The study population was specifically curated to include enough patients with or without clinically significant fibrosis (≥stage 2), advanced fibrosis (≥stage 3) or cirrhosis (stage 4) to be powered to robustly assess sensitivity and specificity. It is important to note that this does not reflect the prevalence of advanced fibrosis in the general population or even routine clinic populations, and the sensitivity and specificity data from this study will be used to confirm the predictive values of the biomarker panels for their intended uses in the final qualification step in stage 2 of NIMBLE. The current analysis included aliquots from a serum sample obtained within 180 d of an evaluable liver biopsy demonstrating NAFLD. For FibroMeter VCTE, a liver stiffness measurement was required within 180 d of the biopsy. Exclusion criteria included pregnancy at the time of sample collection or biopsy, comorbid liver diseases, use of drugs known to cause steatosis, non-availability of minimum required serum, bariatric surgery within 3 years before biopsy, prior liver transplant and known primary or secondary malignancy of the liver.

#### Biomarker panels tested and their intended context of use

Serum biomarker panels selected by the NIMBLE circulating workstream were reviewed and approved by the project team, NASH CRN ancillary study and steering committees and accepted by the FDA in the letter of intent for their qualification. These included:

NIS4^34^: based on mir34a, hemoglobin A1c, α2-macroglobulin and YKL-40

OWLiver^35^: based on triglyceride species with variable number of saturated fatty acids

ELF test^36^: based on type III procollagen peptide, hyaluronic acid and TIMP-1

PROC3^37^: procollagen-3 fragment reflective of fibrogenesis

FibroMeter VCTE^38^: based on liver stiffness measurement by VCTE, age, gender, α2-macroglobulin, international normalized ratio (INR), platelet count, AST and gamma-glutamyl transferase (GGT)

The intended use of NIS4 was to diagnose at-risk NASH and its components, whereas the OWLiver panelsʼ intended use was to diagnose the presence of NASH (Extended Data Table 2). The intended uses of the ELF test, PROC3 and FibroMeter VCTE were to diagnose clinically significant fibrosis (≥stage 2 fibrosis), advanced fibrosis (≥stage 3 fibrosis) or cirrhosis (stage 4 fibrosis).

#### Study approach

The study plan was summarized in a letter of intent approved by the FDA^14,39^. De-identified, barcoded, frozen aliquots of the same serum sample from each participant without any prior freeze–thaw were released to the individual laboratories. These laboratories contemporaneously generated panel scores, which were provided to the independent statistical team (Cytel), which deposited these in the CRN data warehouse. The CRN then released the meta-data linked to the barcodes to Cytel, which implemented the prespecified statistical analysis plan without involvement of individual vendors whose panels were tested. The NIMBLE circulating workstream and statistical team then jointly reviewed the results and interpreted the data.

#### Histological examination

The pathology committee of the NASH CRN performed the histological assessment, masked to clinical and laboratory data, using an established and validated protocol^30,31^. The key measures included the presence of steatohepatitis and individual severity grades for steatosis (0–3), lobular inflammation (0–2), hepatocellular ballooning (0–2) and fibrosis stage (0–4). The NAS was computed from the scores for steatosis, ballooning and inflammation, whereas ‘at-risk’ NASH was computed from the presence of its components^31,39^.

### Statistical analyses

Two pre-specified performance metrics formed the basis for hypothesis testing. First, that the AUROC for each panel would be 0.7 or higher for its intended use with 95% confidence limits that would not intersect 0.5. Next, the biomarker performance would be superior to commonly used blood-based laboratory aids for their intended use. The AUROC of each panel was, therefore, compared to that of ALT for diagnosis of NASH or NAS ≥4 and FIB-4, a commonly used laboratory aid based on age, AST, ALT and platelet counts, for diagnosis of fibrosis severity^19^. The rationale for the use of ALT as a marker of liver injury is that it has been used by the general medical community for this purpose for many decades despite its limitations in the context of NAFLD. FIB-4 was used as the comparator for fibrosis because it is a widely available test that does not require special testing. Although VCTE is widely used in clinical practice, it is not approved by the FDA or the European Medicines Agency as a diagnostic tool for any stage of fibrosis. It was, therefore, not permissible to use it as the comparator, and a VCTE-based test, FibroMeter VCTE, was one of the panels being tested. The imaging workstream of NIMBLE will separately report on VCTE and other ultrasound-based tools and also magnetic resonance imaging (MRI)-based measures. It is important to note that, if the biomarker panels could not outperform these very simple tools, they would not move forward with additional qualification efforts. The sensitivity and specificity were computed at the Youden cutpoint. The sensitivity was further estimated, keeping specificity fixed at 90%, and, conversely, specificity was measured, keeping the sensitivity fixed at 90%. Finally, the PPVs and NPVs were computed at various prevalence of specific NAFLD phenotypes. Missing data were assumed to be missing at random from the statistical analysis, as they resulted from sample handling and laboratory issues independent of the relationship between biomarkers and histology; complete case analysis was done.

### Reporting summary

Further information on research design is available in the Nature Portfolio Reporting Summary linked to this article.

## Online content

Any methods, additional references, Nature Portfolio reporting summaries, source data, extended data, supplementary information, acknowledgements, peer review information; details of author contributions and competing interests; and statements of data and code availability are available at 10.1038/s41591-023-02539-6.

### Supplementary information

Supplementary Information List of IRB protocol approval numbers per study and site

Reporting Summary

## Figures and Tables

**Extended Data Table 1:**
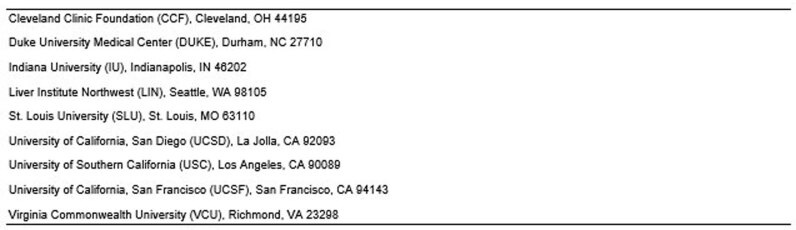
List of participating NASH CRN sites

**Extended Data Table 2:**
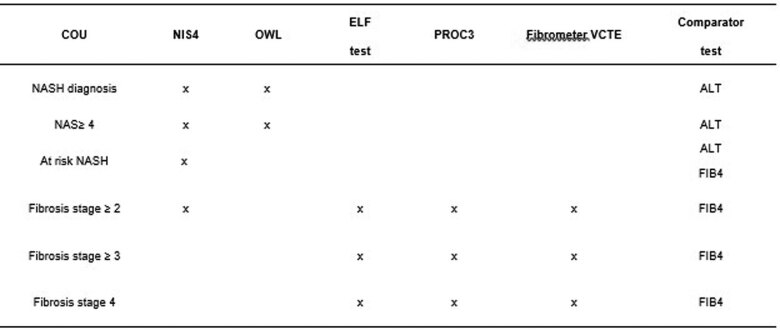
Intended use of biomarker panels and comparators used

**Extended Data Table 3:**
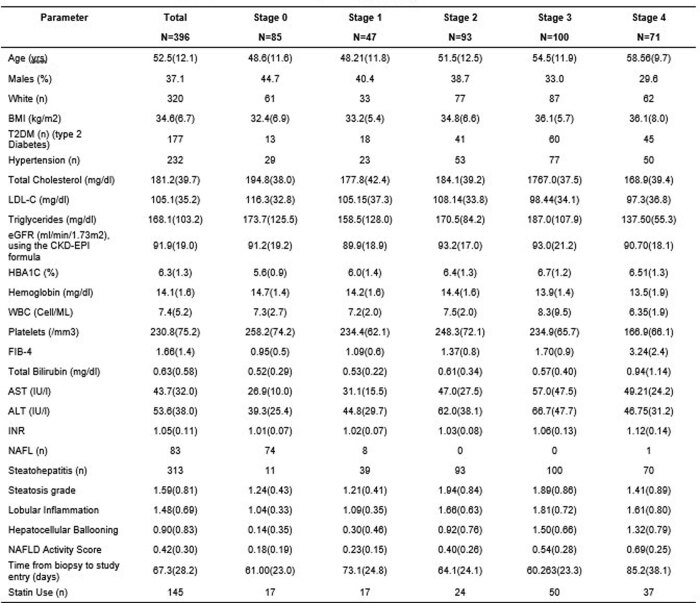
Baseline demographics, clinical and laboratory data for the FibroMeter VCTE cohort

**Extended Data Table 4:**
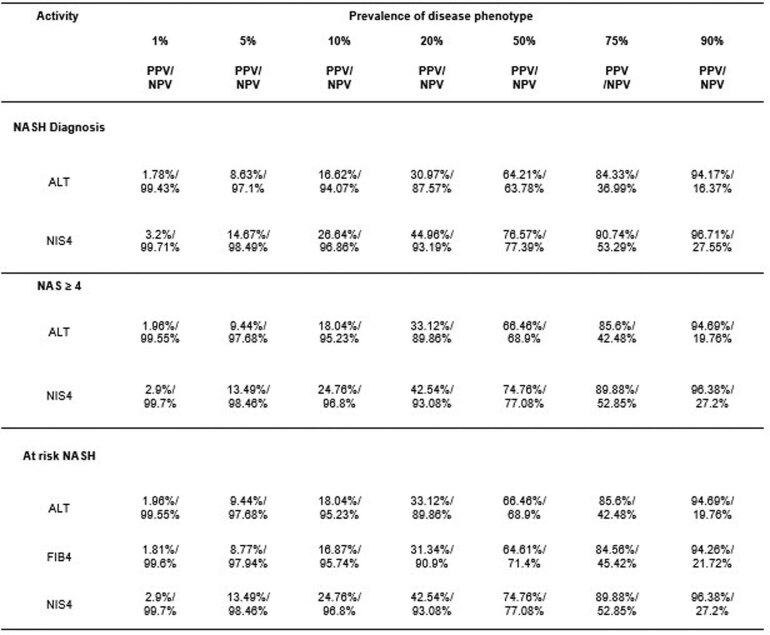
Predictive values of biomarker panels for their intended use in populations with varying prevalence of disease phenotype

**Extended Data Table 5:**
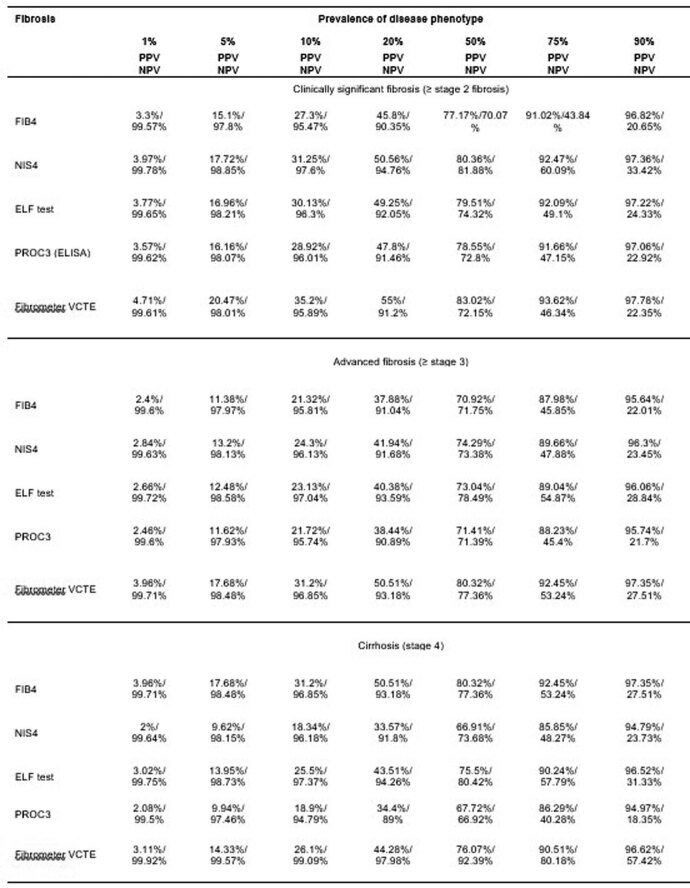
Predictive values of biomarker panels for their intended use in populations with varying prevalence of disease phenotype

## Data Availability

Pre-existing data access policies for each of the parent cohort studies specify that research data requests can be submitted to each steering committee; these will be reviewed promptly for confidentiality or intellectual property restrictions and will not be unreasonably refused. Individual-level patient or assay data may be further restricted by consent, confidentiality or privacy laws and considerations. These policies apply to both the non-publicly available clinical and the assay data. The NAFLD Database, PIVENS and FLINT clinical data are publicly available at the NIDDK Central Repository: https://repository.niddk.nih.gov/home/; the NAFLD DB2 clinical data will be submitted by end of 2023.

## Code availability

The code for the analyses is available on GitHub (project title: NIMBLE circulating workstream stage 1 collaboration with NASH CRN; https://github.com/kcpyates2023/NIMBLE-collaboration-with-NASHCRN). See file with instructions on how to use the codes.
