# Supplementary material for: Diagnostic performance of circulating biomarkers for non-alcoholic steatohepatitis
Source: Nat Med. 2023 Sep 7;29(10):2656–64. doi: 10.1038/s41591-023-02539-6 (PMC10579051; doi:10.1038/s41591-023-02539-6)
Supplement: Supplementary file 1 — List of IRB protocol approval numbers per study and site [file 41591_2023_2539_MOESM1_ESM.pdf]

nature medicine

Article https://doi.org/10.1038/s41591-023-02539-6

Diagnostic performance of circulating biomarkers for non-alcoholic steatohepatitis

In the format provided by the authors and unedited

Supplemental Table 1: List of IRB protocol approval numbers per study and site

Study-site	IRB protocol approval number
**NAFLD Database 2**	IRB# 03-00127
CWRU	IRB# 06-928
CCF	IRB# 06-928
DUKE	Pro#00020074
IU	IRB# 0910-24
SLU	IRB# 16331
SMC	IRB# 5638S-14
UCSD	IRB# 110960
UCSF	IRB# 10-00834
Fresno	IRB# 2012028
VMMC	IRB# IRB07033
VCU	IRB HM12525
DCC	IRB# 00002391
**FLINT**	
CWRU	IRB10-01461
CCF	IRB# 11-032
DUKE	PRO00027848
IU	1101-20
SLU	IRB#: 17084
UCSD	#110081
UCSF	IRB# 10-03940
Fresno	IRB# 2012029
CPMC	2011.008-2
VMMC	IRB# IRB10149
VCU	IRB# HM13416
DCC	IRB# 00003341
**PIVENS**	
CWRU/CCF	IRB#03-00128/ #06-056
DUKE	PRO00009323
IU	0402-34
SLU	IRB# 13167
UCSD	#050362
UCSF	H124-24610-05
UW	IRB0703A
VCU	IRB# 3617
DCC	IRB# H.34.04.02.13.D1
**NAFLD Database**	
CWRU	03127-M-03
DUKE	IRB# 5814-04-5R0ER
IU	IRB# 0405-24
SLU	IRB#: 13170
UCSD	#030509
UCSF	H124-25060-01
UW	04-0678-G/E01
VCU	IRB# 3670
DCC	#H.34.02.03.06.C1
