# Supplementary material for: Diagnostic performance of circulating biomarkers for non-alcoholic steatohepatitis
Source: Nat Med. 2023 Sep 7;29(10):2656–64. doi: 10.1038/s41591-023-02539-6 (PMC10579051; doi:10.1038/s41591-023-02539-6)
Supplement: Supplementary file 2 — Reporting Summary [file 41591_2023_2539_MOESM2_ESM.pdf]

nature portfolio

Corresponding author(s): Arun J. Sanyal

Last updated by author(s): Jul 30, 2023

# Reporting Summary

Nature Portfolio wishes to improve the reproducibility of the work that we publish. This form provides structure for consistency and transparency in reporting. For further information on Nature Portfolio policies, see our Editorial Policies and the Editorial Policy Checklist.

## Statistics

For all statistical analyses, confirm that the following items are present in the figure legend, table legend, main text, or Methods section.

| n/a | Confirmed | |
|---|---|---|
| ☐ | ☒ | The exact sample size (*n*) for each experimental group/condition, given as a discrete number and unit of measurement |
| ☐ | ☒ | A statement on whether measurements were taken from distinct samples or whether the same sample was measured repeatedly |
| ☐ | ☒ | The statistical test(s) used AND whether they are one- or two-sided<br>*Only common tests should be described solely by name; describe more complex techniques in the Methods section.* |
| ☐ | ☒ | A description of all covariates tested |
| ☐ | ☒ | A description of any assumptions or corrections, such as tests of normality and adjustment for multiple comparisons |
| ☐ | ☒ | A full description of the statistical parameters including central tendency (e.g. means) or other basic estimates (e.g. regression coefficient) AND variation (e.g. standard deviation) or associated estimates of uncertainty (e.g. confidence intervals) |
| ☐ | ☒ | For null hypothesis testing, the test statistic (e.g. *F*, *t*, *r*) with confidence intervals, effect sizes, degrees of freedom and *P* value noted<br>*Give P values as exact values whenever suitable.* |
| ☒ | ☐ | For Bayesian analysis, information on the choice of priors and Markov chain Monte Carlo settings |
| ☒ | ☐ | For hierarchical and complex designs, identification of the appropriate level for tests and full reporting of outcomes |
| ☒ | ☐ | Estimates of effect sizes (e.g. Cohen's *d*, Pearson's *r*), indicating how they were calculated |

*Our web collection on statistics for biologists contains articles on many of the points above.*

## Software and code

Policy information about availability of computer code

| | |
|---|---|
| Data collection | Data were collected in clinical record forms from clinical sites of NASH CRN and uploaded to NASH CRN Data Coordinating Center. The meta data was extracted from this and sent to Cytel in an Excel Sheet. Raw data from individual labs for biomarkers were sent directly to Cytel in Excel sheets. These were combined for the analyses. |
| Data analysis | the codes are made available. Please see code availability statement |

For manuscripts utilizing custom algorithms or software that are central to the research but not yet described in published literature, software must be made available to editors and reviewers. We strongly encourage code deposition in a community repository (e.g. GitHub). See the Nature Portfolio guidelines for submitting code & software for further information.

## Data

Policy information about availability of data

All manuscripts must include a data availability statement. This statement should provide the following information, where applicable:

- Accession codes, unique identifiers, or web links for publicly available datasets
- A description of any restrictions on data availability
- For clinical datasets or third party data, please ensure that the statement adheres to our policy

Pre-existing data access policies for each of the parent cohort studies specify that research data requests can be submitted to each steering committee; these will be reviewed promptly for confidentiality or intellectual property restrictions and will not unreasonably be refused. Individual level patient or assay data may further



March 2021

be restricted by consent, confidentiality or privacy laws/considerations. These policies apply to both the non-publicly available clinical and the assay data. The NAFLD DB, PIVENS and FLINT clinical data are publicly available at the NIDDK Central Repository: https://repository.niddk.nih.gov/home/ ; the NAFLD DB2 clinical data will be submitted by the summer 2023.

## Human research participants

Policy information about studies involving human research participants and Sex and Gender in Research.

| | |
|---|---|
| Reporting on sex and gender | these are provided (Table 1) |
| Population characteristics | provided in Table 1 and in Extended data table 3 |
| Recruitment | this is a curated data set from the NASH CRN with relatively balanced distribution of fibrosis stages to avoid spectrum bias |
| Ethics oversight | all studies were IRB approved (see Supplemental Table 1) |

Note that full information on the approval of the study protocol must also be provided in the manuscript.

# Field-specific reporting

Please select the one below that is the best fit for your research. If you are not sure, read the appropriate sections before making your selection.

☒ Life sciences ☐ Behavioural & social sciences ☐ Ecological, evolutionary & environmental sciences

For a reference copy of the document with all sections, see nature.com/documents/nr-reporting-summary-flat.pdf

# Life sciences study design

All studies must disclose on these points even when the disclosure is negative.

| | |
|---|---|
| Sample size | see statistical section. It was powered to identify at risk NASH or advanced fibrosis or cirrhosis with an AUROC 0.8 with 80% power. It is powered for both sensitivity and specificity to ensure rule in and rule out data are robust. |
| Data exclusions | N/A |
| Replication | N/A. |
| Randomization | N/A |
| Blinding | All clinical investigators and the laboratory investigators/staff were blinded to histology and meta data associated with individual samples or even aggregate data. The independent statistical core at Cytel had all of the data and executed the pre-specified statistical plan |

# Reporting for specific materials, systems and methods

We require information from authors about some types of materials, experimental systems and methods used in many studies. Here, indicate whether each material, system or method listed is relevant to your study. If you are not sure if a list item applies to your research, read the appropriate section before selecting a response.

## Materials & experimental systems

| n/a | Involved in the study | |
|---|---|---|
| ☒ | ☐ | Antibodies |
| ☒ | ☐ | Eukaryotic cell lines |
| ☒ | ☐ | Palaeontology and archaeology |
| ☒ | ☐ | Animals and other organisms |
| ☐ | ☒ | Clinical data |
| ☒ | ☐ | Dual use research of concern |

## Methods

| n/a | Involved in the study | |
|---|---|---|
| ☒ | ☐ | ChIP-seq |
| ☒ | ☐ | Flow cytometry |
| ☒ | ☐ | MRI-based neuroimaging |

# Clinical data

Policy information about clinical studies

All manuscripts should comply with the ICMJE guidelines for publication of clinical research and a completed CONSORT checklist must be included with all submissions.

| | |
|---|---|
| Clinical trial registration | NCT# 01030484, 01265498, 00063622 |
| Study protocol | provided as supplemental files |
| Data collection | these are noted in the manuscript and in TRIPOD statement provided as supplemental document |
| Outcomes | N/A |
